# Phase-reference monitoring in coherent-state discrimination assisted by a photon-number resolving detector

**DOI:** 10.1038/srep26025

**Published:** 2016-05-18

**Authors:** Matteo Bina, Alessia Allevi, Maria Bondani, Stefano Olivares

**Affiliations:** 1 Department of Physics, University of Milan, via Celoria 16, I-20133 Milano, Italy; 2 Department of Science and High Technology, University of Insubria and CNISM UdR Como, Via Valleggio 11, 22100 Como, Italy; 3 Institute for Photonics and Nanotechnologies, CNR, and CNISM UdR Como, Via Valleggio 11, 22100 Como, Italy; 4 INFN Sezione di Milano, I-20133 Milano, Italy

## Abstract

Phase estimation represents a crucial challenge in many fields of Physics, ranging from Quantum Metrology to Quantum Information Processing. This task is usually pursued by means of interferometric schemes, in which the choice of the input states and of the detection apparatus is aimed at minimizing the uncertainty in the estimation of the relative phase between the inputs. State discrimination protocols in communication channels with coherent states also require the monitoring of the optical phase. Therefore, the problem of phase estimation is relevant to face the issue of coherent states discrimination. Here we consider a quasi-optimal Kennedy-like receiver, based on the interference of two coherent signals, to be discriminated, with a reference local oscillator. By means of the Bayesian processing of a small amount of data drawn from the outputs of the shot-by-shot discrimination protocol, we demonstrate the achievement of the minimum uncertainty in phase estimation, also in the presence of uniform phase noise. Moreover, we show that the use of photon-number resolving detectors in the receiver improves the phase-estimation strategy, especially with respect to the usually employed on/off detectors. From the experimental point of view, this comparison is realized by employing hybrid photodetectors.

In a phase-estimation protocol, the probe signal is prepared in an optimized pure state that undergoes an unknown phase shift. The phase-shifted signal is then sent to a receiver that retrieves information about the phase by implementing a suitable detection scheme^1^. The goal is to reach the minimum uncertainty allowed by the scheme, i.e. the inverse of the Fisher information (FI), or, in the best case, the minimum uncertainty allowed by quantum mechanics, i.e. the inverse of the quantum FI^2^.

In a binary phase-shift-keyed (BPSK) communication channel a *π* phase shift is usually imposed or not to an input coherent state |*β*〉, thus encoding the logical bits “1” or “0” into |*β*〉 or |−*β*〉, respectively. Thereafter, the problem is to discriminate between the two nonorthogonal coherent states with the minimum error probability, which is theoretically given by the Helstrom bound^1^. During the last decade, many solutions, based either on homodyne detection, photon-number resolving (PNR) detectors or hybrid receivers have been theoretically^3^,^4^,^5^ and experimentally proposed^6^,^7^,^8^,^9^,^10^,^11^. Typically, to discriminate among two or more coherent states each signal interferes at a beam splitter (BS) with a reference coherent state, the local oscillator (LO), whose phase should be known and well-defined. Therefore, both the initial channel calibration and the control of the LO phase are crucial^12^.

One of the main limitations in the realization of this kind of interferometric receivers comes from phase-noise sources, which can affect the generation and propagation of the signals^13^,^14^. It is worth noting that the discrimination must be carried out shot by shot, without any *a-priori* knowledge of the transmitted signal. This requires the assumption that, after the initial calibration, the LO phase always remains fixed. To monitor the phase, the communication must be interrupted and a probe state must be sent to estimate the phase minimizing its uncertainty. Indeed, sometimes the interruption could be unnecessary, namely, when the scheme is still well-calibrated: in this case an “embedded”, “not-disturbing” method would be desirable.

Motivated by the renewed interest in coherent channels for their fundamental relevance to discrimination and estimation theory and also as a resource for deep-space communication^15^, in this paper we investigate whether and at which extent it is possible to retrieve some information about possible shifts of the LO phase without interrupting the communication. To this aim, we process sets of data drawn from the same collected outputs used for the discrimination. This is one of the few attempts, to our knowledge, to deal with estimation by exploiting a setup aimed at state discrimination. Furthermore, the protocol allows avoiding unnecessary losses of information: if the channel is well-calibrated the communication is still going on, otherwise, one knows which data should be discarded before recalibration. Since the discrimination performance of coherent-state receivers has been thoroughly investigated^16^,^17^, in this paper we only focus on the phase estimation. Here, we give a proof of principle of a real-time phase-monitoring protocol involving a Kennedy-like receiver^18^,^19^,^20^. The implementation of this kind of discrimination scheme is less complex than, e.g., the Dolinar receiver^21^ and it could be useful in many practical situations. At variance with the typical configuration of a Kennedy-like receiver, which employs on/off detectors, in this paper we also consider an enhanced version, equipped with PNR detectors. We show that a suitable analysis of the outcomes allows monitoring the LO phase down to the limit imposed by the FI.

## Results

### Error probability, overall input signal and phase estimation

Let us assume that the coherent states |±*β*〉 are sent through the BPSK communication channel with prior probability *z*_+_ = *z*_−_ = 1/2, which maximizes the channel capacity. To achieve quasi-optimal discrimination^3^ we address a receiver in which the signal is mixed at a BS of transmittance *τ* with a LO excited in the coherent state |*α*〉 (*α*, 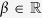 and *α*, *β* > 0). By setting 
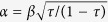
 we have the following mapping: 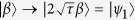 and 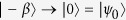. The operative strategy turns out to be the discrimination between the presence and the absence of light, which corresponds to the projective measure 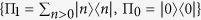. In this case the use of on/off detectors, without any photon-number discrimination power, is sufficient. In the limit *τ* → 1, the error probability 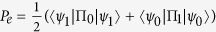 in the discrimination is 
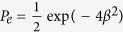
, i.e. twice the minimum error probability given by the Helstrom bound when 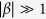
1. The previous treatment assumes that the LO phase *ϕ* is constant and precisely known (in the present case *ϕ* = 0). By choosing the LO excited in the coherent state |*α*e^i*ϕ*^〉 and still in the limit *τ* → 1, the error probability reads


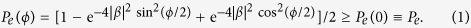


As mentioned in the Introduction, the crucial point now is to monitor the actual value of *ϕ*.

In order to estimate a possible phase shift *ϕ*, our strategy exploits sets of data drawn from the discrimination outputs, previously collected shot by shot. The overall state reaching the receiver is described by the state:


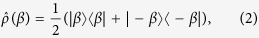


which represents a phase-sensitive balanced mixture of two input signals (unbalanced mixtures will be considered later on). In our scheme the input 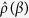 and the LO |*α*e^i*ϕ*^〉 are mixed at a BS with generic *τ*. By defining 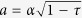 and 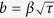, the state after the BS is 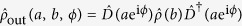, where 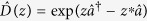 is the displacement operator and 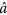 is the annihilation operator, 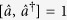. The corresponding photon-number distribution reads:


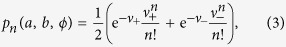


which is the sum of two Poisson distributions depending on *ϕ* through the mean values *ν*_±_ = *a*^2^ + *b*^2^ ± 2*ab* cos *ϕ*. Equation (3) characterizes the statistics of the collected outputs of the discrimination protocol. Therefore, the estimation of *ϕ* and, thus, the real-time phase monitoring, is attainable directly from Eq. (3) by using sets of acquired data and a suitable estimation strategy, as described in Sect. “Methods”. It is worth noting that the presence of losses, characterized by an amplitude damping Γ ∈ [0, 1], just rescales the coherent signal amplitudes 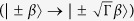 and, accordingly, 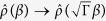. Since the phase is left unchanged, the phase monitoring is still achievable from Eq. (3) upon the substitution 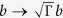. Thus, without loss of generality, in the following we will consider the ideal scenario with Γ = 1 (no amplitude damping).

### Numerical simulations

**Figure 1:**
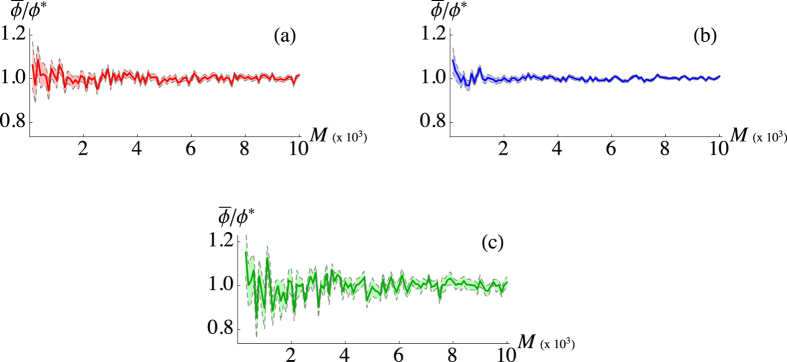
Plots of the ratio 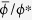 (solid lines) and the corresponding standard deviation (dashed) as functions of *M* using Bayesian strategies with on/off (**a**) and PNR (**b**) detectors, and the inversion of the Fano factor (**c**). Simulated setup parameters: 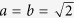 and ϕ* = 0.3.

To test our approach, we firstly performed Monte Carlo simulated experiments. We randomly generated one of the two coherent states |±*β*〉 and recorded the shot-by-shot output (number of detected photons) of the receiver. This information can be used for the shot-by-shot discrimination described above. However, here we do not address the discrimination performance, but we are interested in its phase estimation capability, as mentioned in the Introduction. In particular, we consider sets of the recorded data, whose elements are distributed according to the probability in Eq. (3), corresponding to the output state 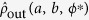, where ϕ* is the actual value of the phase. In general, given a sample {*x*}, of size *M*, and the corresponding probability distribution *p*(*x*|*ϕ*) dependent on the parameter *ϕ*, classical estimation theory provides the optimal limit for an unbiased estimator of the parameter *ϕ* in terms of the Fisher Information (FI) 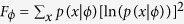, which imposes a lower bound for the attainable variance, namely the Cramér-Rao bound 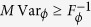. In our analysis we applied the Bayesian method to both on/off and PNR detection and compared the corresponding estimators 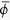, which are asymptotically optimal, i.e. saturate the Cramér-Rao bound^22^, as discussed in Sect. “Methods” [see Fig. 1(a,b)]. Note that the employment of a PNR detector brings 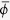 to converge to ϕ* more rapidly, just after a data sample of size *M* ~ 10^3^, than using the on/off detector.

The Bayesian method is very powerful as it converges fast and, as we will see, it provides a result robust against phase noise. Nonetheless, information about the phase can also be retrieved in other ways. For instance, the Fano factor of 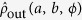 can be directly obtained from the photon distribution measured by the PNR detector. This quantity displays an explicit dependence on *ϕ* and can be inverted to obtain it^23^. By analyzing the same simulated data used before, we can compare this inversion method with the Bayesian one. In particular, Fig. 1(c) shows the phase estimations and the corresponding standard deviation as functions of the sample size *M*, with the same parameters employed for the Bayesian estimation. The plot clearly shows a slower convergence to ϕ*, with very large fluctuations due to error propagation.

**Figure 2:**
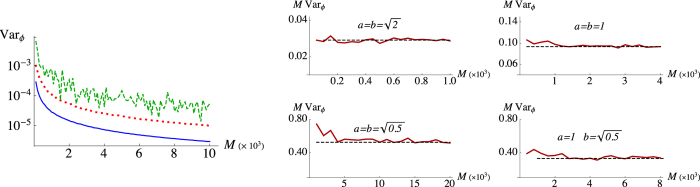
Left: logarithmic plots of Var_*ϕ*_ as functions of *M*, with ϕ* = 0.3 and 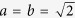, using on/off (red dotted lines) and PNR (blue solid lines) detection, and the inversion of the Fano factor (green dashed line). Right: logarithmic plots of *M* Var_*ϕ*_ (red solid lines), in the case PNR detectors are employed, for different values of the signal and LO energies, compared with 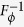 (black dashed lines), i.e. the asymptotic saturation of the Cramér-Rao bound. Convergence, in terms of the number of data *M*, is clearly ruled by the different horizontal axis scales. Note that the higher the energy of the input states, the faster the converge is.

In Fig. 2 (left) we compare Var_*ϕ*_ obtained with the Bayesian strategies and the inversion of the Fano factor, as functions of *M*. The convergence to ϕ* is clearly faster for Bayesian strategies and the use of PNR detector results to be the best strategy since more information can be extracted from the reconstruction of the photon distribution. In the right panel of Fig. 2 we show how the convergence depends on the energy of the input states, especially in the small mean photon number regime. In particular, the higher the mean photon number of the input coherent states (signal and LO), the faster the convergence to the Cramér-Rao bound 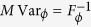 is.

#### Uniform phase noise

**Figure 3:**
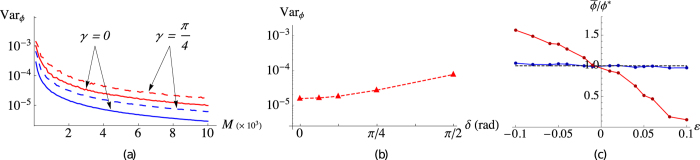
(**a**) Logarithmic plots of Var_*ϕ*_ as functions of *M*, with ϕ* = 0.3 and 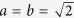, using on/off (red lines) and PNR (blue lines) detection. Comparison of the Bayesian methods without noise *γ* = 0 (solid lines) and with noise *γ* = *π*/4 (dashed lines). (**b**) Logarithmic plot of Var_*ϕ*_ as a function of the LO noise parameter *δ*. (**c**) Plot of the ratio 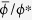 as a function of the unbalancing parameter *ε*. Red (blue) points refer to simulations in the case of on/off (PNR) detection. Simulated setup parameters for figures (**b**,**c**): 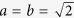, ϕ* = 0.3, *γ* = *π*/4 and *M* = 4000.

In a more realistic scenario the signals can be affected by noise during generation and propagation, which here we simply model by introducing uniform phase noise. This is more detrimental than, e.g., phase diffusion, but it can be better controlled in our experimental setup. The degraded state is the so-called bracket state 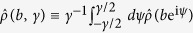, where *γ* is the noise parameter^23^. We now apply the same Bayesian approach by considering the photon-number distribution 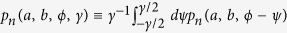 of the output state 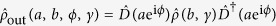. The Bayesian strategies prove to be very robust, only showing small differences in the convergence (Fig. 3(a)) compared to the ideal case (*γ* = 0). As expected, the noise increases the variance in the estimation procedure.

In the presence of a uniform phase noise affecting also the LO phase the photon-number distribution *p*_*n*_(*a*, *b*, *ϕ*, *γ*) should be substituted by 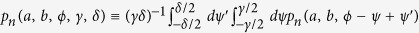, *δ* being the LO noise parameter. The effect of this further noise on phase estimation is shown in Fig. 3(b), where we plot Var_*ϕ*_ as a function of *δ* in the case of a bracket state with *γ* = *π*/4 and for fixed *M* = 4000. As expected, the presence of this further noise worsens the phase estimation. However, for the present choice of the setup parameters, the effect of the noise on the LO becomes relevant for 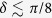. As we noticed above, without loss of generality we can neglect the presence of amplitude damping, since this affects only the coherent-state amplitudes but preserves their phases (however, it will affect the phase estimation convergence rate).

#### Unbalanced mixture of signals

Let us also discuss the case in which our strategy is applied to a data sample corresponding to an *unbalanced* mixture 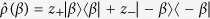 with *z*_±_ = 0.5 ± *ε*, where the unbalancing parameter *ε* ∈ [−0.5, 0.5] represents the deviation from the balanced-mixture condition. We use the Bayesian estimation with a balanced mixture since we assume complete lack of knowledge about the amount of the deviation *ε*. In Fig. 3(c) we show our Monte Carlo simulated results. As one may expect, in both cases (on/off and PNR detectors) the estimator becomes biased. In particular if *ε* < 0 one has 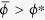, whereas for *ε* > 0 we obtain 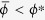. Nevertheless, it is evident from the simulations that phase estimation based on PNR detection is more robust to unbalanced mixtures. Since in general a finite data sample is indeed unbalanced, our results show that PNR detection should be preferred to the on/off counterpart.

### Experimental results

**Figure 4:**
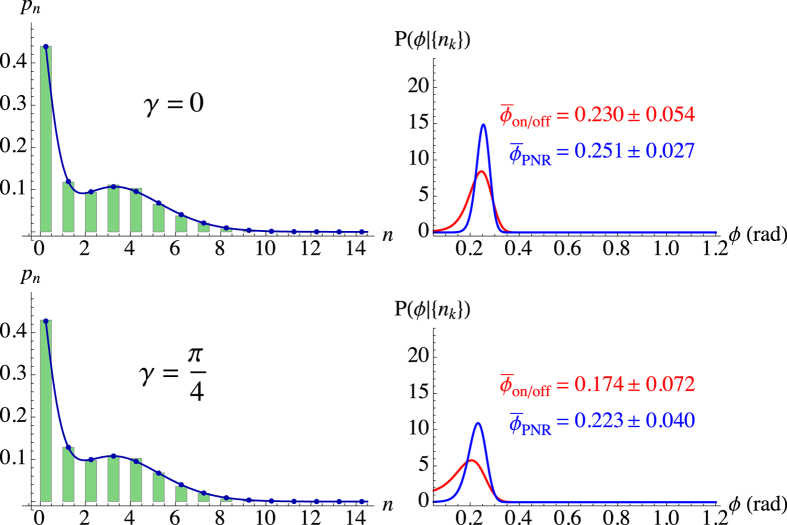
Left: experimental 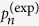 obtained by PNR detection (green histograms) and theoretical expectations 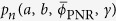 (blue lines). Right: posterior probabilities of *ϕ* for *M* = 4000 with on/off (red curve) and PNR (blue curve) detection. The experimental parameters are *a* = 1.12, *b* = 0.79, 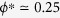, *γ* = 0 (top row) and *γ* = *π*/4 (bottom row).

**Figure 5:**
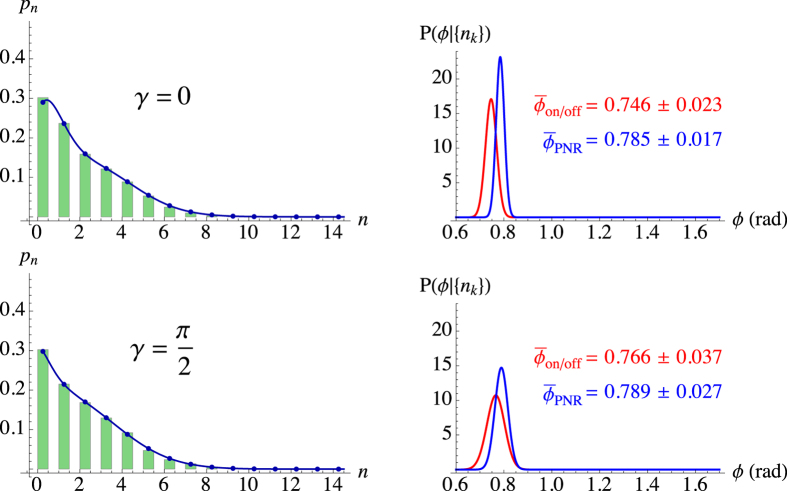
Left: experimental 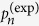 obtained by PNR detection (green histograms) and theoretical expectations 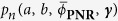 (blue lines). Right: posterior probabilities of *ϕ* for *M* = 4000 with on/off (red curve) and PNR (blue curve) detection. The experimental parameters are *a* = 1.12, *b* = 0.79, 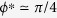, *γ* = 0 (top row) and *γ* = *π*/2 (bottom row).

To experimentally validate the previous analysis about phase shifts, we realized a proof-of-principle scheme (see Sect. “Methods” for details). We realized an experimental scheme in which linearly-polarized laser pulses at 523 nm were sent to a Mach-Zehnder interferometer. The relative phase between the two arms was controlled by a piezoelectric movement operated step by step^24^. The mean number of photons of the output state (see Figs 4 and 5) was chosen in order to match the operational range of the employed detector, which is a hybrid photodetector, whose output was amplified and synchronously integrated. The bracket state 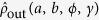 was generated in post selection by combining a set of data having phases in the interval (ϕ* − *γ*/2, ϕ* + *γ*/2) with a second set with phases in the interval (ϕ* + *π* − *γ*/2, ϕ* + *π* + *γ*/2).

In the following we consider the states corresponding to two choices of ϕ*, namely, 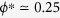 and 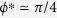 with *γ* = *π*/4 and *γ* = *π*/2, respectively. The results are presented in Figs 4 and 5, where we plot the measured photon-number distribution 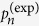 (left panels) and the posterior probabilities *P*_on/off_(*ϕ*|{*n*_*k*_}) and *P*_PNR_(*ϕ*|{*n*_*k*_}) (right panels) with the corresponding estimated phases. To assess the quality of our reconstructions, we also plot the distributions 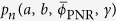, which have fidelities 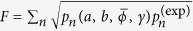 higher than 99.9% to the experimental data^25^. In both Figures it is evident that the PNR detector provides an accurate reconstruction of 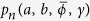 for the two bracket states.

If we focus on the first experimental test concerning 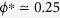 (Fig. 4), we can see that the posterior probabilities, affected by a bias, show an asymmetric shape which is more marked in the case of on/off detection (Fig. 4, right panels). In the case of small phases and a limited amount of data, the optimal estimator is the mode 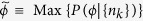. For the considered value 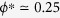, we find that the best phase estimation is 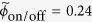, 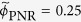 (for *γ* = 0) and 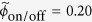, 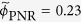 (for *γ* = *π*/4). Strikingly, PNR detection enhances the estimation as the posterior probability is more peaked, has smaller variance and reduced asymmetry. The second experimental test for 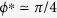 (Fig. 5) displays better performances, in agreement with the expected behavior of FI (see Sect. “Methods”). PNR detection, again, provides an enhancement in the phase estimation that can be described in terms of a smaller variance and a more peaked posterior probability. In both situations, we note that the effect of the phase noise is to broaden the posterior probabilities by increasing Var_*ϕ*_, in agreement with the simulated data and the plot in Fig. 2. Nonetheless, the Bayesian strategy turns out to be very robust in the presence of uniform phase noise with large amplitudes such as *γ* = *π*/4 (Fig. 4, second row) and *γ* = *π*/2 (Fig. 5, second row).

## Discussion

In this paper we have shown that it is possible to perform a useful phase estimation, typically implemented with pure, optimized input probe states, also with mixed, not optimized, probe states, namely the overall mixture of two coherent states. The choice of this state is twofold. On the one hand such a state is the correct way to represent a shot-by-shot classical communication signal encoded in two coherent states with opposite phases, on the other hand the balanced mixture maximizes the binary channel capacity.

Our strategy, based on Bayesian analysis, does not require any interruptions of the communication and asymptotically reaches the minimum uncertainty in the estimation just after few thousands of data. Furthermore, we have demonstrated the advantages of using PNR detectors with respect to on/off detectors and we have tested the performance of our strategy also in the presence of uniform phase noise. We completed our numerical investigation considering a further phase noise on the LO and small deviations from the ideal input balanced mixture, showing the robustness of the Bayesian strategy in phase estimation. Finally, we have provided the experimental proof of principle of the protocol, strengthening our theoretical model and numerical simulations. It is worth noting that the presence of amplitude damping effects preserves the phase of coherent signals but it would affect the convergence rate of phase estimation, i.e. the less the detected number of photons the slower the Bayesian convergence.

Our results not only represent a first step toward making the Kennedy-like receiver a practical useful technology, but they also pave the way to the monitoring of the phase reference in more complicated communication systems or quantum optics setups, which require a precise control of the phase, e.g. in the generation of nonclassical states, such as squeezed states.

## Methods

### Experimental setup

**Figure 6:**
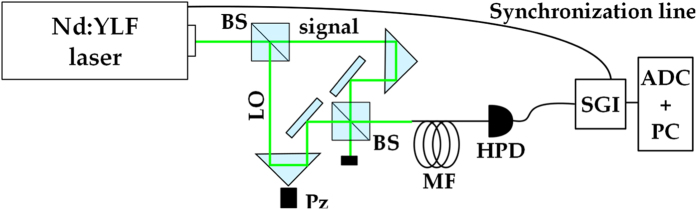
Sketch of the experimental setup. The laser beam is sent to a Mach-Zehnder interferometer, in which the relative phase between the LO and the signal is changed by means of a piezoelectric movement (Pz). One output of the interferometer is delivered to a hybrid photodetector (HPD) by means of a multimode fiber (MF). The amplified output of the detector is synchronously integrated (SGI), digitized (ADC) and processed offline (PC).

The experimental setup used to estimate the phase shifts is sketched in Fig. 6. The second-harmonic pulses (~5-ps pulse duration) at 523 nm of a mode-locked Nd:YLF laser regeneratively amplified at 500 Hz (High-Q Laser Production) are sent to a Mach-Zehnder interferometer, in which the relative phase between the two arms (signal and LO in Fig. 6) is changed in steps by means of a piezoelectric movement. In particular, 320 different values of phase *ϕ* are considered and for each of them a suitable data sample is saved.

The light exiting the interferometer is delivered through a multi-mode fiber (600-*μ*m-core diameter) to a commercial photon-number-resolving detector exploiting a hybrid technology (HPD, R10467U-40, maximum quantum efficiency ~0.5 at 500 nm, 1.4-ns response time, Hamamatsu). It is essentially a photomultiplier, in which the second stage of amplification is performed by a diode operated below the breakdown threshold. This detector has the advantage of operating at room temperature (in our case its temperature is stabilized at 16 °C by means of a water chiller) and can be operated within the visible spectral range. The output of the detector is amplified (preamplifier A250 plus amplifier A275, Amptek), synchronously integrated over a 500-ns window (SGI, SR250, Stanford) and digitized (AT-MIO-16E-1, National Instruments). We notice that in such a detection apparatus the dark-count contribution can be neglected: in the absence of light, no peak corresponding to one detected photon is present in the pulse-height spectrum.

As already explained in some previous works^24^,^26^,^27^, with our detection system we are able not only to reconstruct the statistics of detected photons, and hence the number of detected photons, but also to determine the actual value of the phase *ϕ* at each piezo position, independent of the regularity and reproducibility of the movement. The procedure used to achieve this goal is made possible by the linearity of HPD. By monitoring the mean number of detected photons as a function of the piezolelectric movement, an interference pattern emerges from which the value of *ϕ* can be estimated^24^.

Once the relative phase is determined, the simulation of bracket states with a uniform phase distribution can be achieved, in post selection, by combining a set of data characterized by a central phase *ϕ* and a uniform integration interval *γ* and appending it to a second set corresponding to an interval with the same amplitude but with opposite phase. In order to approach the operation of a real communication channel, a numerical randomization of data in the bracket states, followed by an estimation algorithm, is performed. We notice that, in principle, the same can be experimentally achieved by randomizing the piezoelectric movement, even if spurious effects such as the hysteresis of the device can limit the performance of the system. However, it is worth noting that the randomization is not fundamental in our protocol, since the Bayesian analysis does not require it: the posterior Bayesian probability depends on the data sample, but not on the order of the data.

Finally, a possible limitation to an effective signal discrimination is given by the fringe visibility, which in our proof-of-principle experiment is more than 90%. Indeed, all these parameters must be optimized and taken into account in a real communication channel, which is beyond the aim of our investigation.

### Bayesian estimation

To monitor the phase, we extract from the raw outcomes (in the case of a PNR detector, a string of integer numbers corresponding to the detected number of photons) a sample of size *M*, namely, {*n*_*k*_} = {*n*_1_, *n*_2_, …, *n*_*M*_}, 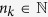, 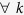. This can be done while the communication proceeds. As {*n*_*k*_} implicitly depends on *ϕ*, we build the sample probability 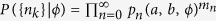, being *p*_*n*_(*a*, *b*, *ϕ*) the photon-number distribution defined in Eq. 3, and *m*_*n*_ the number of occurrences of *n* detected photons, so that 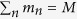. *P*({*n*_*k*_}|*ϕ*) is thus the probability of obtaining the sample given *ϕ*. Due to Bayes theorem, we can write the posterior probability of *ϕ* given the sample, namely, 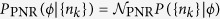, where 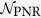 is a normalization factor and *ϕ* is assumed to have uniform prior distribution. The Bayes estimator of *ϕ* is 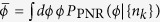 and its variance is 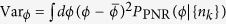. This estimator is asymptotically optimal^28^,^29^, that is 
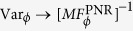
 if 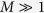, where 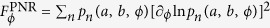 is the FI associated with *p*_*n*_(*a*, *b*, *ϕ*). It is now clear that by selecting consecutive samples {*n*_*k*_} we can monitor the phase value throughout the whole communication, so that we can check the channel calibration, and also be aware of the data to be possibly discarded. Without this phase tracking scheme, one should periodically stop the communication to check the calibration: if the channel is found to be not calibrated, there is no chance to know at which point the received signals have been processed by using the wrong phase and, thus, which data should be discarded.

**Figure 7:**
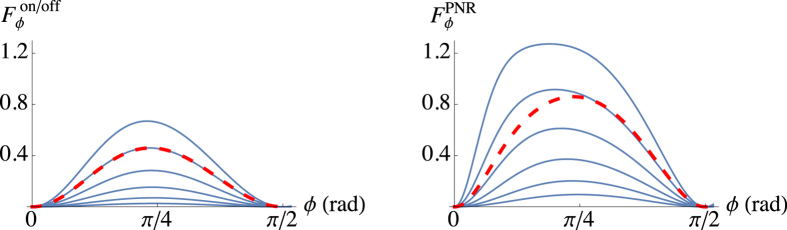
FI for on/off (left) and PNR (right) detection as a function of *ϕ* for *a* = *b* = 0.5, 0.6, 0.7, 0.8, 0.9, 1.0 (blue lines, from bottom to top). Note the higher values of 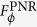 with respect to 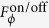. The red dashed lines refer to our experimental configuration (*a* = 1.12 and *b* = 0.79).

Since on/off detection can be seen as a particular case of PNR detection, its Bayes estimator is obtained straightforwardly. Given the same sample {*n*_*k*_} considered above and defining *P*_off_ ≡ *p*_0_(*a*, *b*, *ϕ*) and 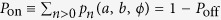, the sample probability reduces to 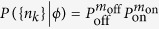, where *m*_off_ and *m*_on_ = *M* − *m*_off_ are the number of “off” (*n*_*k*_ = 0) and “on” (*n*_*k*_ > 0) events, respectively. The posterior probability is thus given by *P*_on/off_(*ϕ*|{*n*_*k*_}) = *N*_on/off_*P*({*n*_*k*_}|*ϕ*). Also in this case the Bayes estimator asymptotically reaches the optimal value, where the corresponding FI is now 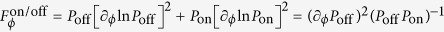. In Fig. 7 we plot the FI for on/off and PNR detection, which displays a maximum in the interval (0, *π*/2), and it vanishes at *ϕ* = 0, *π*/2. It is worth noting that in both on/off and PNR detection the FI goes to zero as *ϕ* → 0: this is a consequence of the probe mixed state 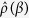 in Eq. (2), which is a balanced mixture of the state |*β*〉, which maximizes the FI for *ϕ* = 0, and |−*β*〉, that leads to a null FI for *ϕ* = 0. As one may expect, since the receiver is aimed at dealing with state discrimination and not with phase estimation, the FI is much smaller than the quantum FI (see below).

### Quantum Fisher information

The ultimate limit to the precision of a parameter estimation is imposed by a generalized Cramér-Rao bound Var_*ϕ*_ ≥ [*M* *H*_*ϕ*_]^−1^, where *H*_*ϕ*_ is the quantum Fisher information (QFI), which is the maximum information that can be extracted from a quantum system as it maximizes the FI over all possible types of measurement, such that *H*_*ϕ*_ ≥ *F*_*ϕ*_.

Here we evaluate the QFI for the parameter *ϕ* starting from the overall output state:


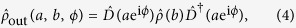


where 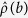 is given in Eq. (2) of Sect. “Results” and the parameter to be estimated is *ϕ*. We assume *a*, 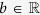. It is worth noting that 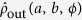 depends on the parameter *ϕ* through the generator 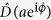, that is not the usual phase shift operator 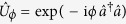. Upon introducing the two parameters *λ*_1_ ≡ *λ*_1_(*a*, *ϕ*) = *a* cos *ϕ* and *λ*_2_ ≡ *λ*_2_(*a*, *ϕ*) = *a* sin *ϕ*, we can rewrite the displacement operator as:


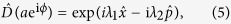


where 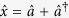 and 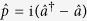. Therefore, the quantity of interest is now a function *ϕ*(*λ*_1_, *λ*_2_) of the parameters just introduced. The problem of estimating *ϕ* (at a fixed value of *a*) is turned into a multi parametric estimation and the ultimate precision on the estimation of *ϕ* is thus given by (we drop the statistical scaling)^2^:


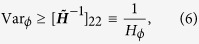


where 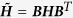 is a 2 × 2 real matrix, ***H*** is the QFI matrix of the estimation of *λ*_1_ and *λ*_2_ and:


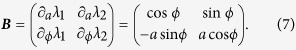


If we expand 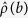 in terms of its eigenbasis, namely, 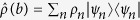, the elements of the QFI matrix ***H*** can be calculated as follows^30^:


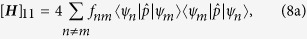



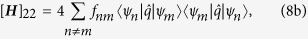



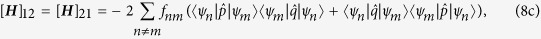


with *f*_*nm*_ = *ρ*_*n*_(*ρ*_*n*_ − *ρ*_*m*_)^2^/(*ρ*_*n*_ + *ρ*_*m*_)^2^. Note that ***H*** does not depend on *a* and *ϕ*. The same procedure can be followed to calculate the ultimate bound to the precision in the presence of phase noise by substituting 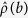 with 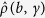.

**Figure 8:**
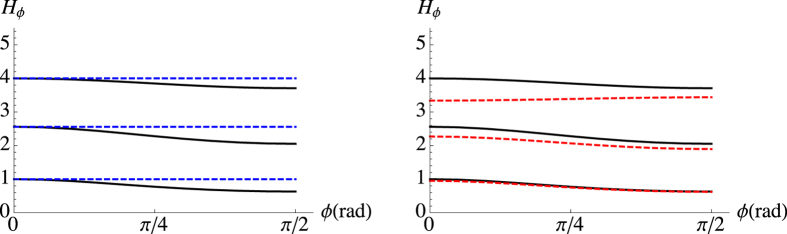
Plot of *H*_*ϕ*_ as a function of the phase *ϕ* for three values of *a* = *b* = *α*: from bottom to top, *α* = 0.5, 0.8, 1.0 (black lines). Left: horizontal blue dashed lines correspond to the values 4|*α*|^2^ of the QFI obtained for a single coherent state |*α*〉 as the probe state. Right: red dashed lines describe the behavior of *H*_*ϕ*_ in the presence of uniform noise with *γ* = *π*/4.

Figure 8 (left) shows *H*_*ϕ*_ as a function of the phase *ϕ* for different values of coherent states amplitudes. We compare the QFI of the output state 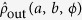 when the input state 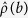 is the mixture in Eq. (2) (black solid lines) and when the input is the single coherent state |*b*〉 (blue dashed lines), which maximizes the QFI. As one may expect, *H*_*ϕ*_ is much larger compared to the Fisher information *F*_*ϕ*_ achieved by the Kennedy receiver (for comparison see Fig. 7). In Fig. 8 (right) we compare the previous results in the presence of uniform noise: the higher the energy the more detrimental the effect of noise.

## Additional Information

**How to cite this article**: Bina, M. *et al.* Phase-reference monitoring in coherent-state discrimination assisted by a photon-number resolving detector. *Sci. Rep.* **6**, 26025; doi: 10.1038/srep26025 (2016).
